# Management of Chronic Non-Cancer Pain Through a Multicomponent Workshop Based on Non-Pharmacological Therapies: A Protocol for a Mixed-Methods Study, Including a Two-Arm Parallel-Group Randomized Clinical Trial and a Qualitative Component

**DOI:** 10.3390/healthcare14183063

**Published:** 2026-09-17

**Authors:** María Victoria Ruiz-Romero, Rosa Anastasia Garrido-Alfaro, Almudena Arroyo-Rodríguez, María Blanca Martínez-Monrobé, Consuelo Pereira-Delgado, Ángela C. López-Tarrida, Juan V. Luciano, José Manuel López-Millán, Serafín Moro-Muñoz, María Dolores Guerra-Martín, Patricia Pérez-García, María Begoña Gómez-Hernández

**Affiliations:** 1 Hospital San Juan de Dios del Aljarafe, 41930 Bormujos, Spain; mariavictoria.ruiz@sjd.es (M.V.R.-R.);; 2 San Juan de Dios Foundation, 20036 Madrid, Spain; aarroyor@comillas.edu (A.A.-R.);; 3 Health Sciences Department, San Juan de Dios School, Comillas Pontifical University, 41930 Bormujos, Spain; 4 Department of Clinical and Health Psychology, Autonomous University of Barcelona, 08193 Bellaterra, Spain; 5 Teaching, Research & Innovation Unit, Parc Sanitari Sant Joan de Déu, 08830 Sant Boi de Llobregat, Spain; 6 Centre for Biomedical Research in Epidemiology and Public Health (CIBERESP), 28029 Madrid, Spain; 7 Anesthesiology, Reanimation and Pain Treatment Service, Virgen Macarena University Hospital, 41009 Seville, Spain; 8 Department of Surgery, University of Seville, 41009 Seville, Spain; 9 Department of Nursing, Faculty of Nursing, Physiotherapy and Podiatry, University of Seville, 41009 Seville, Spain; 10 Fundación para la Gestión de la Investigación en Salud de Sevilla, 41013 Seville, Spain

**Keywords:** chronic pain, non-pharmacological therapies, multicomponent workshop, randomized controlled trial, qualitative research, self-management

## Abstract

**Highlights:**

**What does this protocol contribute?**

- This protocol describes a mixed-methods study comprising a two-arm parallel-group randomized controlled trial to evaluate a structured multicomponent workshop based on non-pharmacological therapies for chronic non-cancer pain, together with a qualitative component.
- This study is designed to assess the effectiveness, acceptability, and perceived usefulness of the intervention, while exploring how participants incorporate the strategies learned into daily life.

**What are the potential implications?**

- This study may provide evidence to support the integration of structured non-pharmacological interventions into person-centered chronic pain care.
- The intervention may be transferable to other healthcare settings, including primary care and pain units.

**Abstract:**

**Background/Objectives**: Chronic non-cancer pain often requires approaches beyond pharmacological treatment. This protocol describes a mixed-methods study evaluating the effectiveness of a multicomponent intervention based on non-pharmacological therapies (NPhTs) and exploring participants’ post-intervention experience. **Methods**: The design includes a randomized, controlled, two-arm parallel-group clinical trial with 1:1 allocation, embedded within a mixed-methods approach that includes a qualitative phenomenological strand. This study will be conducted at Hospital San Juan de Dios del Aljarafe, Spain. A total of 160 adults with chronic non-cancer pain will be randomly assigned to the intervention or control group. The intervention group will receive five weekly 3.5 h face-to-face sessions combining pain education, emotional regulation, cognitive-motivational and mind–body techniques, lifestyle recommendations, peer support, and home practice, supported by a mobile application. The control group will continue usual care, without the workshop or intervention-related app content. The primary outcome will be change in the EuroQol-5D (EQ-5D) index from baseline to 1 month. Secondary outcomes will include the EQ-5D visual analogue scale (VAS) score, pain intensity, well-being, self-esteem, resilience, anxiety and depression, pain catastrophizing, medication use, and healthcare resource use. Outcomes will also be assessed at 4 months and, in the intervention group, at 7 months. Semi-structured interviews will explore the participants’ experiences, perceived usefulness of the techniques, and barriers and facilitators to maintaining the strategies learned. **Conclusions**: This protocol will evaluate the effectiveness and acceptability of a structured group-based NPhT intervention for chronic non-cancer pain. The findings may inform its applicability to person-centered and self-care-oriented models of care. **Trial registration:** ClinicalTrials.gov NCT06440668.

## 1. Introduction

Chronic pain (CP) is defined as pain that persists or recurs for longer than 3 months or extends beyond the expected period of tissue healing [1]. It can be classified as chronic primary pain, in which pain constitutes a health condition itself, or chronic secondary pain, in which pain occurs in the context of an underlying condition [2].

CP affects an estimated 20–30% of adults worldwide [3,4,5], and 25.9% in Spain [6]. It has been associated with female sex, older age, lower educational level, unemployment, and greater healthcare resource use [7]. Consistent with the ICF framework, CP may involve impairments in body functions and, where relevant, structures, limitations in daily activities, and restrictions in social and occupational participation [8], alongside lower quality of life (QoL) [6]. The higher prevalence observed among women [6,7] supports considering sex and gender when examining the psychosocial impact of CP, coping strategies, and barriers to self-management.

Although pharmacological treatment remains an important component of CP management, it may be insufficient as a standalone approach and can involve adverse effects and adherence problems, particularly with long-term opioid use [9,10,11]. Current approaches, therefore, increasingly integrate pharmacological and non-pharmacological interventions within multimodal models of care [10,12,13,14].

Within these models, person-centered care considers patients’ experiences, needs, and preferences and promotes active participation in care, while self-management involves using skills and strategies to manage the physical, emotional, and everyday consequences of CP [13,14]. Non-pharmacological therapies (NPhTs) encompass physical, psychological, cognitive, mind–body, and lifestyle-related strategies that can support these goals by addressing different dimensions of CP [15]. NPhTs may be combined within multicomponent and multidisciplinary interventions [12,16].

### 1.1. Background

Multicomponent interventions combining several NPhTs have reported generally favorable pain-related, functional, and psychosocial outcomes in chronic non-cancer pain [16,17,18]. Evidence from multidisciplinary biopsychosocial rehabilitation for chronic low back pain suggests modest effects on pain and disability compared with usual care [19], while heterogeneity in intervention components, study designs, and follow-up periods limits conclusions regarding the effectiveness of specific programs [16]. The clinical relevance of these effects should, therefore, be interpreted cautiously.

The present study focuses on adults with chronic non-cancer pain, including both chronic primary and chronic secondary pain. Hospital San Juan de Dios del Aljarafe has delivered a multicomponent NPhT-based workshop for this population since 2016, using a standardized format since November 2021. The program consists of five weekly 3.5 h face-to-face sessions, integrating pain education, emotional regulation, cognitive and motivational strategies, mind–body techniques, lifestyle-related self-care, peer contributions, and home-based practice, with the aim of promoting active patient participation and the continued use of self-management strategies in daily life. Peer interaction is intended to promote shared learning, mutual support, and engagement with self-management strategies [20]. Previous qualitative and quasi-experimental evaluations of the workshop suggested improvements across pain-related, psychological, and QoL outcomes, as well as medication use and healthcare resource utilization [21,22,23]. However, these studies were conducted by the same research group within the same program and used non-randomized designs, limiting causal inference and leaving uncertainty regarding comparative effectiveness under randomized allocation and the longer-term maintenance of observed changes.

The present protocol addresses these uncertainties through a mixed-methods, randomized controlled trial. Random allocation will allow a more robust assessment of comparative effectiveness, while additional follow-up of the intervention group will explore longer-term outcome maintenance. Adherence will be monitored through session attendance, and a mobile application will support activity recording and continued self-management practice during follow-up, consistent with previous evidence on digital self-management support for chronic pain [24,25]. The qualitative strand will complement the trial by exploring the participants’ experiences, perceived usefulness of the intervention components, and personal or contextual factors that facilitate or hinder their incorporation into daily life—dimensions that cannot be fully captured through standardized outcome measures.

### 1.2. Aims

#### 1.2.1. Primary Aim

The primary aim of this project is to evaluate the effectiveness of a multicomponent NPhT-based workshop in improving health-related quality of life in adults with chronic non-cancer pain.

#### 1.2.2. Secondary Aims

The secondary aims are to:

- Evaluate the effect of the intervention on complementary measures of health-related QoL, pain intensity, well-being, self-esteem, mood, resilience, anxiety and depression, pain catastrophizing, medication use, and healthcare resource use, including emergency department visits and scheduled consultations.
- Explore patients’ experiences and perceptions of the workshop, including its perceived influence on pain, daily life, participation in meaningful activities, and future goals, using qualitative methodology based on semi-structured interviews.
- Assess overall satisfaction with the workshop using an ad hoc questionnaire.
- Identify which techniques and tools presented and practiced during the workshop are most helpful for pain management and well-being, using qualitative methodology and an ad hoc questionnaire.
- Identify patient-related factors and characteristics associated with greater or lower workshop success, using both quantitative and qualitative methods.

## 2. Study Protocol

### 2.1. Design and Methodology

#### 2.1.1. Design

This study uses a quantitatively driven embedded mixed-methods design with predominantly sequential timing.

The quantitative strand constitutes the core component of this study and consists of a two-arm parallel-group randomized controlled trial and a 1:1 allocation ratio, designed to evaluate intervention effectiveness. The comparative period will last 4 months from the baseline assessment. The intervention group will also be followed for 7 months.

The qualitative strand is embedded within the overall study and is conducted after completion of the intervention to explore the participants’ lived experiences, perceived usefulness of the techniques, and personal and contextual factors influencing their incorporation into daily life.

Priority will, therefore, be given to the quantitative strand, while the qualitative findings will provide complementary and contextual information that cannot be fully captured through standardized outcome measures. Integration will occur at the interpretation and reporting stages by examining convergence, divergence, and complementarity between quantitative and qualitative findings and, where appropriate, through joint displays.

Due to the nature of the intervention, participants and intervention providers cannot be blinded; only the researcher responsible for analyzing the results will be blinded. The research support technician will be responsible for collecting the results and extracting them from the platform. The “blinded researcher” will be responsible solely for data analysis and will not have access to the platform. The groups will be coded as A and B, and each patient will be assigned a numerical code linked to their identifying information in a separate database that will not be accessible to the researcher conducting the statistical analysis.

#### 2.1.2. Setting

This study will be conducted at Hospital San Juan de Dios del Aljarafe, a regional hospital located in Andalusia, Spain, which serves a catchment population of approximately 303,000 inhabitants. The intervention will be delivered face-to-face at the hospital facilities.

#### 2.1.3. Intervention

The participants allocated to the intervention group will receive a multicomponent psychoeducational workshop focused on self-management, pain control, and emotional regulation, based on NPhTs.

The participants will continue to receive their usual healthcare. Participation in the workshop will not require discontinuation or mandatory modification of analgesic medication, psychotropic drugs, or any other usual treatments. Any reduction, withdrawal, or modification of pharmacological treatment will occur, where applicable, at the patient’s decision and/or according to the judgement of the healthcare professionals responsible for their usual clinical care. Current pharmacological treatment will be recorded both at baseline and at the different follow-up time points (months 1, 4, and 7, with the latter only in the intervention group), as well as whether the participants are receiving physiotherapy, psychological treatment, or complementary therapies (specifying which ones); whether they engage in regular physical exercise (specifying the type and frequency); whether they are currently receiving care from a pain management unit; and whether they use healthcare services due to pain (emergency department visits, scheduled outpatient visits, and hospitalizations). This information will allow us to document changes in the healthcare received over the course of this study.

The workshop will follow the standardized structure used in previous editions of the program. A detailed session-by-session description of the workshop is provided in Appendix A (Table A1). The intervention is also described in detail elsewhere [23]. Briefly, the intervention will consist of five weekly face-to-face group sessions, each lasting 3.5 h. The sessions will combine pain education, emotional regulation strategies, cognitive and motivational exercises, mind–body techniques, lifestyle-related self-care recommendations, peer testimony, and home-based practice. The workshop will be delivered by professionals with specific training and experience in CP management, including preventive medicine, rehabilitation medicine, internal medicine, nursing, psychology, and physiotherapy. The instructors will remain the same throughout this study, as will their presentations and teaching materials. The instructors have at least four years of experience delivering these workshops. Over this period, they have refined their interventions in consultation and agreement with the workshop coordinator (the Principal Investigator of this study), resulting in standardized content, delivery methods, and materials provided to the participants, all of which will remain unchanged throughout the study. The patients who previously completed the workshop may also participate as peer contributors by sharing their experiences of coping and applying the tools learned.

During the workshop, participants will be given tasks to practice at home between sessions, which they will be asked to record daily.

All of the participants will have access to specific MoviSalud mobile app (version 3.21.5; Socioemprende S.L., Valencia, Spain), features to complete questionnaires (baseline and follow-up) in both groups (control and intervention). The MoviSalud platform currently supports the management of a range of clinical conditions across specialties such as Cardiology, Internal Medicine, and Pulmonology, among others, and is available for use in both public and private hospitals following its purchase. This platform was selected because of its previous experience in hospital settings and the high level of satisfaction reported by healthcare professionals who have used it. It will be used primarily for data collection. Only in the intervention group will it also be used to support follow-up, as selected materials (audio recordings of the main meditation exercises, exercise guides, and an explanatory video on chronic pain) will be uploaded to the application. These materials will serve as reminders and reinforcement to help patients continue applying the tools practiced during the workshop. These materials will become available upon completion of the workshop and will remain accessible until the end of the follow-up period (month 7). Only the participants in the intervention group will have access to these intervention-related digital contents.

During the first session, a messaging group will be created for each workshop and coordinated by a volunteer patient. The group will be used to share reinforcement materials from the sessions, channel questions to the workshop director, and communicate attendance-related incidents. It will also provide a space for peer support, helping to strengthen group cohesion and a sense of belonging during the intervention.

The final session will include workshop closure, a review of the tools learned, and participants’ final assessment of the workshop. The participants will also receive a guide reinforcing the content covered during the workshop. Access to the MoviSalud mobile app will be maintained so that the participants can continue applying the tools and recommendations after the workshop and complete the follow-up assessments.

Adherence to the intervention will be monitored through session-attendance records. The participants attending at least four of the five workshop sessions will be classified as adherent for the per-protocol sensitivity analysis. Completion of the outcome assessments will be recorded separately and will not determine adherence status or inclusion in the intention-to-treat population. The participants who discontinue or attend fewer than four sessions will continue to be invited to complete the scheduled follow-up assessments unless they withdraw consent.

The intervention groups from the different workshop sessions will not communicate with one another and will not know each other. The participants in the control groups will not communicate in any way with the other study participants; they will only have contact with the research support technician who provides assistance with the app.

#### 2.1.4. Comparator

The participants allocated to the control group will continue to receive the usual care and treatment prescribed by their healthcare professionals during the comparative period. They will not receive the workshop or have access to the support/intervention content of the mobile app during this period. They will have access to the MoviSalud platform only to complete the study questionnaires and records at the scheduled assessment time points.

After completion of the 4-month follow-up, the participants in the control group will be offered the opportunity to attend the workshop under the same protocol. These participants will not be included in the analysis as part of the intervention group.

### 2.2. Sample and Participants

#### 2.2.1. Sample Size

The sample size was determined based on the comparison between the two individually randomized study arms. The original calculation was based on a standardized difference of d = 0.50. A previous assessment of the same workshop population showed a standard deviation of approximately 0.197 for the EQ-5D index. Therefore, d = 0.50 corresponds to an absolute difference of approximately 0.10 EQ-5D index units. This correspondence is provided solely to contextualize the standardized effect used in the original sample-size calculation and is not a decision threshold for intervention effectiveness.

Using G*Power version 3.1.9.6 (Heinrich Heine University Düsseldorf, Düsseldorf, Germany) (two-tailed independent-samples *t*-test, α = 0.05, power = 0.80, 1:1 allocation, and standardized effect size d = 0.50), a total sample size of 128 participants, 64 per arm, was obtained. The complementary calculation for paired samples yielded a smaller sample size requirement and, therefore, did not determine the final sample size.

Because the primary endpoint is assessed at 1 month, the recruitment target was set to protect the primary analysis against the missing primary-outcome data at this time point. Based on previous workshop experience, approximately 20% of the randomized participants may fail to provide the 1-month assessment. Compensating for this anticipated loss requires 160 participants (128/0.80 = 160). To provide an additional operational margin for unanticipated missingness and later follow-up, this study will aim to recruit 180 participants (90 per arm). Later attrition may accumulate over the longer follow-up, but the 4-month and 7-month analyses are secondary or exploratory and do not determine the sample size for the primary endpoint.

Although the intervention is delivered in workshop groups, randomization is performed at the individual level and clustering occurs only in the intervention arm. A conventional cluster-randomized design effect, therefore, will not be applied. No empirical workshop-specific ICC was available to support a formal ICC-based inflation. Potential workshop-level correlation will be addressed prospectively using the prespecified partially nested mixed-effects model. The target of 180 participants provides additional recruitment margin beyond the formal individual-level requirement but is not presented as a formal ICC-adjusted power calculation.

For the qualitative strand, maximum variation sampling will be used to capture the widest possible diversity of perspectives and to identify convergences, patterns of behavior, and common elements in the participants’ experiences. Profiles will be defined according to sex, age, condition, and level of workshop adherence. When possible, at least one participant from each profile will be invited for interview. Including sex as a variation criterion will allow this study to explore potential gender differences in the participants’ experiences, barriers, facilitators, and meanings attributed to CP and the use of self-management strategies. The estimated minimum sample size will be 10 participants. Data collection and analysis will proceed iteratively, and recruitment will continue until data saturation is reached. Saturation will be considered to have been achieved when successive interviews no longer provide new information relevant to the emerging categories and subcategories or reveal new meanings related to the study objectives. Preliminary saturation will be determined by consensus among the researchers involved in the qualitative analysis. Once preliminary saturation has been identified, two additional interviews will be conducted to confirm that no new relevant information emerges. If new relevant information is identified during these interviews, recruitment will continue until saturation is reached and confirmed again.

#### 2.2.2. Participants and Eligibility Criteria

The study population will consist of adults with chronic non-cancer pain receiving care in the catchment area of Hospital San Juan de Dios del Aljarafe.

Chronic non-cancer pain includes the following conditions: fibromyalgia, osteoarthritis, back disorders, joint pain, post-traumatic pain, widespread pain, chronic primary pain, migraine, and autoimmune diseases when they cause musculoskeletal pain. Patients may also have other associated clinical conditions, such as chronic fatigue, provided that these are associated with chronic pain.

The inclusion criteria will be: age between 18 and 70 years; living in the hospital catchment area; diagnosed chronic non-cancer pain treated for at least 6 months, with insufficient response to usual treatment (pain persisting at an intensity of 4 or more on a 0–10 scale, approximately 4 or more days per week); agreement to participate in this study through written informed consent; and completion of the baseline documentation required for the initial assessment.

The exclusion criteria will be: being in the diagnostic or etiological assessment phase of pain; pain exclusively associated with oncological disease; neuropathic pain; a life expectancy of less than 1 year; or severe cognitive or mental disorders that prevent understanding of the workshop content or completion of the measurement instruments.

During the initial interview, an internal medicine physician from the research team will ensure that the patient meets the inclusion criteria and does not meet any exclusion criteria, such as the presence of neuropathic pain. Neuropathic pain will be identified using the Douleur Neuropathique en 4 Questions (DN4) questionnaire [26]. Based on our previous experience, we observed that patients with neuropathic pain do not respond well to these workshops; therefore, these patients will be excluded from the study. In addition, patients will be specifically asked whether they are currently participating in, or plan to participate in, other similar programs or workshops during the study period. If they answer affirmatively, they will not be included in this study.

#### 2.2.3. Recruitment and Randomization

Potentially eligible patients will be identified by physicians from the hospital departments involved in the care of patients with CP, including Rehabilitation, Trauma and Orthopedic Surgery, and Internal Medicine. Patients may also be identified by primary care physicians in the hospital catchment area. These professionals, who provide care to patients with chronic non-cancer pain, are familiar with these workshops and have access to the applicable inclusion and exclusion criteria. When physicians at HSJDA identify a patient who, despite having received usual treatment for at least 6 months, continues to experience pain, the patient’s clinical report is sent to the hospital’s Patient Services Department with a request for inclusion in a workshop. The report is then forwarded by this department to the workshop coordinator. Primary care physicians send the patient’s information in encrypted form via the corporate email system of the Andalusian Public Health System to the workshop coordinator. Patients come from one of the 28 municipalities within the HSJDA catchment area (26 in the province of Seville and 2 in the province of Huelva). These patients are placed on a waiting list in order of referral date.

Each referred patient will be contacted by telephone to confirm eligibility. Eligible patients will receive information about the workshop and this study and will be invited to participate. Those who agree will receive the participant information sheet and informed consent form by email. These documents must be signed and returned before inclusion in this study.

The participants who provide informed consent will be added to the study candidate list in the MoviSalud platform. Subsequently, once a total of 40 patients is reached, the platform will subsequently perform computer-generated random allocation to either the intervention or the control group.

### 2.3. Data

#### 2.3.1. Outcomes

The primary outcome will be the between-group difference in health-related quality of life (QoL), assessed using the EuroQol-5D (EQ-5D) index score [27] at one month, adjusted for baseline values, and reported with its 95% confidence interval. Intervention effectiveness will be evaluated primarily on the basis of this randomized between-group comparison. Improvements greater than 0.10 in the EQ-5D index will be used to aid interpretation of the magnitude of the estimated treatment effect and will not constitute a separate criterion for declaring effectiveness.

The EQ-5D index will also be assessed at 4 months in both randomized groups as a supportive secondary assessment. The additional month-seven assessment in the intervention group will be considered an exploratory evaluation of the maintenance of effect and will not be used for randomized between-group inference because the control group will be offered the workshop after the 4-month comparative period.

Secondary outcomes will include pain intensity, pain catastrophizing, subjective well-being, self-esteem, mood, resilience, anxiety and depression, medication use, health habit improvement, and healthcare resource use. The participant experience-related outcomes will include satisfaction, perceived usefulness of the techniques learned, recommendation of the workshop, perceived impact on daily life, and suggestions for improvement.

The planned outcomes and measurement instruments are summarized in Table 1. The use of validated scales and ad hoc patient-reported outcome and experience measures in previous evaluations of the workshop is described elsewhere [22,23].

For interpretative purposes, previously reported clinically relevant change thresholds will be considered where available: approximately 0.03–0.10 for the EQ-5D index and ≥7–10 points for the EQ-5D visual analogue scale [34,35]; ≥2 points for pain intensity [36]; 2–3 points for self-esteem [37]; 1.5–1.7 points per HADS subscale [38,39]; and approximately 9–11 points for pain catastrophizing [40]. For the well-being and resilience scales, no established and validated minimal clinically important difference (MCID) has been identified. Therefore, in these cases, the effect size of the observed changes will be assessed. The results derived from the ad hoc questionnaires, including questions on mood, health habits, self-perceived improvement in self-esteem, satisfaction measures, and questions regarding perceived impact (effectiveness of the techniques and changes in medication), will be considered exploratory measures, as they are not based on validated scales.

All of the patients (control and intervention groups) will record the following information on the baseline data collection form: medication name, dose, frequency of administration, and rescue medication (if pain is not relieved). They will provide this information again at 1 and 4 months. Based on the information reported by patients at baseline and during follow-up, the number of analgesics and anxiolytics/antidepressants/hypnotics will be recorded at baseline and at each follow-up assessment.

Participant experience-related outcomes will be assessed at the end of the workshop. These will include overall satisfaction, perceived usefulness of the techniques learned, recommendation of the workshop, the techniques considered most useful, and perceptions of the impact of the intervention on pain, health habit improvement, mood, self-esteem and life project, and they will provide a self-assessment of changes in medication (whether there was a reduction in the frequency or dose of analgesics/psychotropic medications, whether they switched to a lower-level medication, or whether they discontinued one or more medications).

Suggestions for improvement and free-text comments will also be collected. These aspects will be assessed using ad hoc questionnaires and semi-structured interviews.

#### 2.3.2. Participant Timeline

The participant timeline is summarized in Table 2. After eligibility assessment and written informed consent, the participants will complete the baseline assessment and will be randomly allocated to either the intervention group or the control group.

(1) Baseline assessment: All outcomes included in Table 1, except health habits and participant experience. (2) Follow-up assessments: All outcomes included in Table 1, except semi-structured interviews; only the ad hoc questionnaires (satisfaction questionnaire and self-assessment questionnaire on the impact of the workshop). (3) Qualitative study: Semi-structured interviews.

The participants in the intervention group will attend the five-session workshop over 1 month. They will complete follow-up assessments at the end of the workshop (1 month after baseline); 4 months after baseline (corresponding to 3 months after completion of the workshop); and 7 months after baseline (corresponding to 6 months after completion of the workshop).

The participants in the control group will complete assessments at baseline, 1 month after baseline, and 4 months after baseline. After completing the 4-month assessment, the participants in the control group will be offered the opportunity to attend the workshop.

Semi-structured interviews for the qualitative strand will be conducted after completion of the workshop, preferably between the first and second month after the intervention.

This study is expected to last 2 years. Recruitment will take place over 12 months, and follow-up will be completed 18 months after the start of this study. During the final 6 months, data cleaning, analysis, and dissemination will be conducted.

#### 2.3.3. Data Collection

At baseline, sociodemographic and clinical variables will be collected to characterize the sample and explore potential factors associated with response to the intervention. These variables will include age, sex, place of residence, educational level, employment status, family structure, need for a caregiver, caregiver role, pain characteristics, pain duration, pain location, previous pharmacological treatment, previous use of NPhTs, and beliefs about their benefits. Collecting these variables will allow the sample to be described and the results to be contextualized from clinical, social, and gender perspectives.

For the quantitative assessment, the study questionnaires, scales, and surveys completed by the participants will be used. These questionnaires will be embedded in the MoviSalud mobile app, allowing all questionnaires (baseline and follow-up) to be completed and recorded by both groups (control and intervention). Once the participants are enrolled in the study (prior to randomization), they will receive a message explaining how to download the app and access it to enter their baseline data, and they will be provided with a username and password. The participants will be offered telephone support to resolve any questions or technical issues. If a patient has difficulty using the application, they will be asked whether a family member can assist them, in which case support will be provided through that family member. If no such support is available, individualized assistance will be offered either by telephone or in person at the hospital, with an appointment arranged as needed. Reminders will be sent regarding the assessments to be completed at each follow-up time point, and the application will enable communication between patients and healthcare professionals to resolve questions and issues.

For the qualitative assessment, in-depth semi-structured interviews will be conducted following an interview guide developed for this study. The interviews will be audio-recorded and subsequently transcribed for analysis. They will be conducted by nurses with experience in qualitative methodology, care provision, and CP management. These researchers will not be involved in delivering the workshop, to help participants express their experiences, views, and perceptions of the intervention freely. The semi-structured interview guide is included as Appendix B.

The research team will be able to use the MoviSalud professional portal to monitor completion of the planned questionnaires and records. They will also be able to resolve incidents related to use of the app during the data collection process.

#### 2.3.4. Data Management

The study data will be managed using the MoviSalud RPM (Remote Patient Monitoring) platform, adapted for this project. The platform will centralize data entered by the participants through the mobile app and will allow the research team to access these data through a professional web portal.

An internal medicine physician from the research team will conduct recruitment through a telephone interview to verify whether the patient meets the inclusion criteria. If eligible, the physician will inform the patient about this study and the possibility of being allocated to either the intervention or the control group and will obtain informed consent. Once a cohort of 40 patients has been recruited, the physician will notify the research support technician, who will register and enroll them in the app.

Once enrolled in the platform, the patient will have access exclusively to the baseline assessment forms prior to randomization. Once patients have completed the baseline assessment, randomization will be requested. Treatment allocation cannot be predicted in advance.

Allocation to the study arm will be performed automatically by the system through an atomic and irreversible transaction, with a timestamp and audit trail; no individual will be involved in the allocation process. The recruitment wave will then be closed, the corresponding workshop cohort will be established, and the control group will be activated. Within each combination of recruitment wave and stratum, permuted blocks of size 4 or 6 will be generated, with the block size selected randomly and independently for each block, with a probability of 0.50 for each size. With recruitment waves of approximately 40 participants divided into two sex strata, and considering the higher expected prevalence of women among individuals with chronic pain, the male stratum may comprise approximately 10–12 participants per wave. Blocks of size 8 would, therefore, result in a relatively high number of incomplete blocks at the end of each recruitment wave, which would be contrary to the intended purpose of blocking. Block sizes of 4 and 6 limit the maximum imbalance within each stratum to three participants, while preserving allocation unpredictability.

The allocation sequence will be generated using the PCG64 pseudorandom number generator [41], as implemented in the randomization module of the MoviSalud platform (version 3.21.5; Socioemprende S.L., Valencia, Spain), initialized with a cryptographically secure seed. The seed will be encrypted and securely stored, and its SHA-256 hash will be timestamped before the first allocation is issued by the module. This will allow the sequence to be reproduced and verified for audit purposes without making it possible to anticipate future allocations at any time. Independent verification will assess the actual allocation ratio, block lengths, integrity of stratification, uniformity, and absence of serial patterns. The entire process will be documented in a record signed by both responsible individuals. To ensure that methodological decision-making and technical implementation are not assigned to the same individual, these functions will be separated. A methodological lead will define and approve the parameters of the allocation scheme, independently verify the generated sequence before its implementation, and sign the sequence-generation record. A technical lead will configure the module according to the approved specifications, execute sequence generation, safeguard the seed, and ensure technical segregation and traceability. The technical lead will not determine the methodological parameters but will implement those previously approved. Neither individual will participate in patient referral, screening, delivery of the workshop, or outcome assessment.

The platform will support the creation of users, profiles, and differentiated permissions. It will also allow anonymized case management and separate environments for participants in the intervention and control groups. The data will be hosted in a secure cloud environment provided by Amazon Web Services, in accordance with Spanish and European data protection and security regulations. MoviSalud includes authentication mechanisms, access control, secure communication, and protection against unauthorized access.

The study data will be exportable in CSV files for the research team. Once data collection has been completed, the quantitative database will be cleaned in a spreadsheet and then imported for statistical analysis. For the qualitative strand, interviews will be transcribed and checked before data management and analysis.

### 2.4. Ethical Considerations

This study was approved by the Research Ethics Committee of Hospitales Universitarios Virgen Macarena y Virgen del Rocío in October 2025 (SICEIA-2025-002927). The trial is registered at ClinicalTrials.gov under the identifier NCT06440668. Any substantial protocol amendments will be reported to the relevant ethics committee and, where applicable, to the trial registry, in accordance with applicable ethical and regulatory procedures.

All of the participants will receive oral and written information about the study aims, planned procedures, random allocation to study groups, data collection and processing, and the potential benefits and burdens of participation. Participation will be voluntary and will require written informed consent before inclusion in the study. The participants may decline to participate or withdraw from this study at any time by informing the workshop coordinator, without this affecting their usual healthcare.

Personal and clinical data will be treated confidentially and coded to preserve the participants’ identities. Access to information will be restricted to the authorized research team. The results will be analyzed and disseminated in aggregate and anonymized form.

As this study includes a qualitative strand, the participants involved in the qualitative strand will provide additional written informed consent for the semi-structured interviews and for audio recording. The recordings will be transcribed and analyzed while preserving the participants’ confidentiality and anonymity.

The intervention under evaluation is non-pharmacological and is considered low risk. However, participation may involve time burden, questionnaire completion, and possible emotional discomfort when reflecting on the experience of CP. The participants allocated to the control group will be offered the workshop after completion of the comparative follow-up period, to ensure that they are not denied the opportunity to receive a potentially beneficial intervention.

The psychologist on the research team will be available in specific cases in which a patient requires urgent psychological support during the workshop or during the qualitative study interview. After completion of the workshop and/or interview, if the research team considers that a patient would benefit from psychological support, the patient will be advised to seek such support after completing the study.

### 2.5. Data Analysis

#### 2.5.1. Quantitative Analysis

Statistical analysis will be performed using IBM SPSS Statistics, version 27.0 (IBM Corp., Armonk, NY, USA). For categorical variables, absolute frequencies and percentages will be calculated. For quantitative variables, means and standard deviations (SDs) or medians and interquartile ranges will be reported, depending on whether the variables follow a normal distribution. This study has a single confirmatory primary analysis: the baseline-adjusted between-group difference in the EQ-5D index at 1 month, evaluated at a two-sided α level of 0.05. The EQ-5D contrast at 4 months and the prespecified secondary outcomes will be treated as supportive secondary analyses. Analyses of ad hoc outcomes, treatment-effect modification, subgroup analyses, per-protocol analyses, and the month-seven maintenance assessment will be considered exploratory. No global formal adjustment for multiplicity will be applied to these secondary and exploratory analyses because they will not be used to support independent confirmatory claims. Effect estimates and 95% confidence intervals will be emphasized; *p* values for secondary and exploratory analyses will be reported as nominal and interpreted cautiously in the context of multiple testing, and isolated nominal *p* values < 0.05 will not be considered confirmatory evidence.

Continuous secondary outcomes measured repeatedly will be analyzed using analogous linear mixed-effects models. Binary outcomes will be analyzed using logistic generalized linear mixed-effects models; count outcomes will be analyzed using Poisson or negative-binomial mixed-effects models, according to dispersion; and ordinal outcomes will be analyzed using an appropriate ordinal mixed-effects model when applicable.

The primary analysis will follow the intention-to-treat principle. All of the randomized participants will be analyzed in the group to which they were originally assigned, regardless of their adherence to the intervention. The participants attending fewer than four workshop sessions will be classified as non-adherent, but will not be considered lost to follow-up and, unless they withdraw consent, will continue to be invited to complete the scheduled outcome assessments and will remain included in the intention-to-treat population. Intervention discontinuation, adherence, and loss to outcome follow-up will be recorded separately. Longitudinal mixed-effects models will use all available outcome measurements and will be estimated by maximum likelihood under a missing-at-random assumption. Intervention discontinuation, adherence, and loss to outcome follow-up will be recorded separately. If outcome data are missing, multiple imputation under a missing-at-random assumption will be used as a prespecified sensitivity analysis, including treatment allocation, baseline outcome values, available repeated outcome measurements, relevant baseline prognostic variables, and available information related to attendance/adherence and missingness.

When sample size allows, quantitative results will be described, disaggregated by sex, and potential differences in primary and secondary variables will be explored. Sex will be included as a prespecified exploratory effect modifier. For longitudinal outcomes, the treatment × sex and treatment × visit × sex interactions will be assessed. Where the data permit, adjusted intervention effects will be presented separately by sex, with 95% confidence intervals. These analyses will be considered exploratory, as this study was not specifically powered to detect interaction effects.

Given that the participants in the intervention arm receive the intervention in workshop groups, and that all workshop sessions are conducted at the same study center using the same structure and standardized procedures, a workshop-level random effect will be considered within the intervention arm to account for potential residual correlation and heterogeneity across workshop sessions. The structure is partially nested, as the participants in the control group are not assigned to workshops.

The participants who do not complete the 4-month questionnaires will be considered lost to follow-up. Their sociodemographic and clinical characteristics, as well as their baseline scale scores (included in the baseline assessment), will be analyzed to characterize the profile of these patients. If, during follow-up, a participant from either the control or intervention group modifies their medication or the therapies they receive, they will not be considered lost to follow-up; they will remain in this study, and changes will be assessed at 1, 4, and 7 months. The patients originally assigned to the control group will not be included as part of the intervention group in this study. Although they will be offered the opportunity to participate in a workshop after completing their follow-up as controls, they will not be considered part of the intervention group for study purposes.

#### 2.5.2. Qualitative Analysis

The qualitative strand will adopt a descriptive phenomenological approach grounded in Husserlian phenomenology and operationalized according to Giorgi’s descriptive phenomenological method. This approach was selected because the qualitative component aims to describe the participants’ lived experiences following the intervention and to understand the meaning they attribute to the techniques learned and their incorporation into everyday life.

The qualitative analysis will be supported by ATLAS.ti, version 26 (ATLAS.ti Scientific Software Development GmbH, Berlin, Germany).

Following Giorgi’s approach, the analysis will involve: (1) reading each transcript in its entirety to obtain a sense of the whole experience; (2) identifying meaning units relevant to the phenomenon under study; (3) transforming these meaning units into phenomenologically sensitive expressions while remaining grounded in the participants’ accounts; and (4) synthesizing the transformed meaning units to describe the general structure of the participants’ experience. Throughout the analytical process, researchers will adopt a reflexive phenomenological attitude and will seek to bracket prior assumptions regarding chronic pain and the intervention.

Sex/gender will be considered as a cross-cutting interpretive axis in the qualitative analysis. Attention will be paid to differences in the experience of pain, caregiving roles, barriers to self-management, participation in the workshop, and perceived usefulness of the techniques learned.

The analysis will be conducted independently and subsequently discussed by two external researchers who are not involved in the organization or delivery of the workshop.

The qualitative findings will be used to explain the quantitative results and to identify potentially useful information not captured by the scales and questionnaires administered. Integration will take place in Section 3.

### 2.6. Rigor, Validity, and Reliability

The quantitative and qualitative strands of this study were designed and will be reported according to their respective standards: SPIRIT [42] and CONSORT [43] for the randomized trial, and SRQR [44] and COREQ [45] for the qualitative study based on semi-structured interviews. The intervention was described in accordance with the Template for Intervention Description and Replication (TIDieR) checklist [46], which is provided in Supplementary Materials.

The methodological quality of the quantitative strand will be ensured by the use of validated instruments to assess the main study variables. These include the EQ-5D for health-related QoL [27], the Numerical Rating Scale for pain intensity [28], the Rosenberg Self-Esteem Scale [29], the BRS for resilience [30,31], the HADS for anxiety and depression [32], and the PCS for pain catastrophizing [33]. Data collection will be standardized across the predefined assessment time points for both groups during the comparative period. In addition, the intervention will follow a homogeneous structure in terms of sessions and content, and adherence will be monitored through attendance records. To reduce the risk of bias in between-group comparisons, the researcher responsible for the quantitative data analysis will remain blinded to the participants’ allocation.

In the qualitative strand, rigor will be addressed according to Lincoln and Guba’s quality criteria, including credibility, transferability, dependability, and confirmability. Participants with different sociodemographic profiles will be included to capture a sufficient diversity of experiences and perceptions regarding the intervention. The analytical process, including decisions related to coding, categorization, and interpretation, will be documented in detail to enhance the traceability of the analysis and allow it to be reviewed.

## 3. Discussion

This protocol describes a mixed-methods study designed to evaluate a structured multicomponent workshop based on NPhTs for patients with chronic non-cancer pain. This study addresses the need to assess this type of intervention using a more robust design than previous non-randomized evaluations of the program [21]. It also incorporates a qualitative strand to explore participants’ experiences, the perceived usefulness of the techniques, and the factors that may facilitate or hinder their integration into everyday life.

The mixed-methods approach is a strength of this study. The quantitative results will allow comparison between the intervention and control groups during the comparative period. The qualitative data will provide a deeper understanding of how participants perceive the intervention and incorporate the tools learned into their daily lives. Together, these approaches will provide complementary perspectives on the effects of the intervention and participants’ experiences.

The multicomponent and multidisciplinary approach may support the integration of structured NPhTs into person-centered care models oriented towards self-care. In addition, the group-based, face-to-face format may promote peer support and shared learning. These elements are relevant in CP care, where education, emotional regulation, lifestyle-related strategies, and sustained self-care may need to be addressed together rather than as isolated components.

From an operational perspective, this study will require coordination between hospital services and primary care professionals to identify and refer potentially eligible patients. The face-to-face group format also requires appropriate planning of the sessions, the availability of a suitable space, and coordination among the professionals involved. To support intervention fidelity and rigor in the analysis of the results, the workshop will follow a standardized structure and content across the different editions.

Although this study will be conducted in a hospital setting, the intervention does not require complex clinical equipment. However, its implementation requires trained professionals, multidisciplinary coordination, repeated face-to-face sessions, and digital support. These requirements, together with the single-center setting and the specific expertise of the multidisciplinary team, should be considered when assessing transferability. The program may be adaptable to other healthcare settings, such as primary care centers or pain units, although its transferability will need to be evaluated in different organizational and clinical contexts.

The mobile application is conceived as a support tool after the face-to-face workshop. It is intended to reinforce continuity in the use of the techniques learned during the sessions. It is not intended to replace the interpersonal and group-based components of the intervention, but rather to help the participants maintain self-care strategies once the sessions have ended. However, differences in digital literacy, access to suitable mobile technology, and engagement with the application may influence its use, adherence to the digital component, and the completeness of app-based data.

Incorporating a gender perspective may contribute to a more contextualized interpretation of the experience of CP and responses to group-based therapeutic education interventions. The interpretation of effectiveness will be guided primarily by the prespecified primary outcome, while secondary outcomes will provide complementary information on specific domains. Given the number of secondary outcomes, these findings should be interpreted cautiously, considering the magnitude and clinical relevance of the observed changes rather than statistical significance alone.

### Limitations

Several limitations should be considered. First, due to the nature of the intervention, it will not be possible to blind the participants or the professionals delivering the workshop. This may introduce biases related to the participants’ expectations or to the dynamics of the intervention itself. However, the researcher responsible for data analysis will remain blinded to the participants’ allocation. Group interaction and peer support may also influence the participants’ expectations and self-reported responses.

Second, outcomes will be assessed using self-reported measures, including pain intensity, well-being, mood, perceived usefulness of the techniques, and satisfaction with the workshop. Although these measures are appropriate for the study aims, they may be influenced by recall bias, social desirability, or participants’ expectations regarding the intervention.

Third, between-group comparisons will be limited to the first 4 months, as the participants in the control group will be offered the opportunity to attend the workshop after this period. Therefore, the 7-month assessment will only allow maintenance of effects to be explored within the intervention group.

Fourth, the multicomponent nature of the intervention will limit attribution of the observed effects to specific techniques included in the workshop. The intervention combines therapeutic education, professional input, peer interaction, home-based practice, and digital support. The results should, therefore, be interpreted as reflecting the overall effect of the program, rather than the isolated efficacy or relative contribution of its individual components.

Fifth, the clinical heterogeneity of the study population may increase variability in response and limit conclusions regarding specific diagnostic subgroups. At the same time, this heterogeneity reflects the population for whom the workshop is intended in routine practice. Sixth, because the intervention is delivered in successive workshop groups, participants attending the same workshop may show some residual within-group correlation despite individual randomization and standardized intervention delivery. This potential dependence will be addressed analytically using a partially nested mixed-effects structure. However, the limited number of workshop groups may reduce the precision with which the workshop-level variance component can be integrated.

Seventh, analyses of sex-related differences and interactions will be exploratory, as the trial was not specifically powered to assess effect modification by sex and an unequal sex distribution is expected in this population.

Finally, losses to follow-up may occur due to the duration of the workshop, the face-to-face attendance required for the sessions, and repeated questionnaire completion. To reduce this risk, standardized procedures for follow-up and contact with the participants will be applied.

## 4. Conclusions

This protocol describes a mixed-methods randomized controlled clinical trial with a qualitative strand designed to evaluate a multicomponent workshop based on NPhTs for people with chronic non-cancer pain. The study is designed to generate evidence on the effectiveness and to provide complementary information on the participants’ experiences, perceived usefulness, and acceptability-related aspects of a group-based intervention focused on therapeutic education, self-care, and continuity in the use of non-pharmacological strategies. Its findings may contribute to informing interdisciplinary care models, although the transferability of the intervention to other healthcare settings will require further evaluation in different organizational and clinical contexts.

## 5. Intellectual Property

The workshop protocol described in this manuscript is registered as intellectual property under the title “Workshop for the Management of Chronic Non-Cancer Pain with Non-Pharmacological Therapies” [original title in Spanish: “Taller para el Manejo del dolor crónico no oncológico con Terapias no Farmacológicas”]. The registered author is María Victoria Ruiz-Romero. Intellectual Property Registration Number: 202499902031901 (Date of Registration: 1 March 2024).

## Figures and Tables

**Table 1:** Planned outcomes and measurement instruments.

Outcome/Domain	Measurement Instrument or Indicator
Health-related QoL	EuroQol-5D (EQ-5D): index value and visual analogue scale [27]
Pain intensity	0–10 numeric pain rating scale [28]
Subjective well-being	0–10 numeric well-being scale developed within the program [22,23]
Self-esteem	Spanish version of the Rosenberg Self-Esteem Scale [29]
Resilience	Spanish version of the Brief Resilience Scale (BRS) [30,31]
Anxiety and depressive symptoms	Spanish version of the Hospital Anxiety and Depression Scale (HADS) [32]
Pain catastrophizing	Spanish version of the Pain Catastrophizing Scale (PCS) [33]
Mood	Ad hoc categorical item: cheerful, normal, discouraged, or depressed [22,23]
Medication use	Number of medications and changes in analgesic regimen [22,23]
Health habits	Ad hoc items on self-reported habit improvement [22,23]
Healthcare resource use	Number of emergency department visits and scheduled consultations related to CP or to exacerbation or complications of the underlying condition causing pain
Participant experience	Ad hoc questionnaires and semi-structured interviews

**Table 2:** Participant timeline and study procedures.

Timepoint/Period	Timing	Participants Involved	Main Procedures
Screening/inclusion	Before baseline	Potentially eligible patients	Eligibility assessment; participant information; informed consent
T0: Baseline	Month 0	Both groups	Randomization; collection of sociodemographic and clinical variables; outcomes assessment (1)
Intervention period	Month 0–1	Intervention group	Workshop sessions
T1: End of workshop	Month 1	Both groups	Outcomes assessment (2)
		Intervention group	Workshop satisfaction and perceived impact
Qualitative follow-up	Month 2–3	Intervention group	In-depth semi-structured interviews (3)
T2: Follow-up	Month 4	Both groups	Outcomes assessment (2)
		Control group	Offer of the workshop
T3: Follow-up	Month 7	Intervention group	Outcomes maintenance assessment (2)

## Data Availability

No new data were created or analyzed in this study. Data sharing is not applicable to this article.

## Supplementary Materials

The following supporting information can be downloaded at: https://www.mdpi.com/article/10.3390/healthcare14183063/s1, The TIDieR (Template for Intervention Description and Replication) Checklist.

## Abbreviations

The following abbreviations are used in this manuscript:

ANOVA	Analysis of variance
BRS	Brief Resilience Scale
CONSORT	Consolidated Standards of Reporting Trials
COREQ	Consolidated Criteria for Reporting Qualitative Research
CP	Chronic pain
EQ-5D	EuroQol-5D
HADS	Hospital Anxiety and Depression Scale
IASP	International Association for the Study of Pain
MCID	Minimal clinically important difference
NPhT	Non-pharmacological therapy
NPhTs	Non-pharmacological therapies
PCS	Pain Catastrophizing Scale
QoL	Quality of life
RPM	Remote Patient Monitoring
SD	Standard deviation
SPIRIT	Standard Protocol Items: Recommendations for Interventional Trials
SRQR	Standards for Reporting Qualitative Research

## Appendix A

**Table A1:** Workshop content and facilitators.

**Session 1:**
**Objectives:** To explore the participants’ expectations before the workshop; to understand what chronic pain is, how it develops, and how it differs from acute pain; to understand the relationship between physical and emotional pain; to identify limiting beliefs; and to learn the two techniques that participants will practice at home. **Content, techniques, facilitator, duration, and materials:** 1. Welcome and signing of participation and image-use consent forms. (Physician 1) 25 min. 2. Assessment of workshop expectations (brainstorming). (Physician 1) 20 min. 3. Presentation: Pathophysiological mechanisms of pain. Differences between acute and chronic pain. (T: ED) (Physician 2) 60 min. 4. Presentation: Influence of emotions on pain intensity. (T: ED) (Physician 1) 60 min. 5. Activity: My limiting beliefs. (T: TCC) (Physician 1) 15 min. 6. Activity: Mental analgesia technique. (T: meditation) (Physician 1) 15 min. 7. Homework assignments: The participants are instructed to perform the “mirror affirmations to motivate change” and “mental analgesia technique” activities every day and record them on a paper tracking sheet. (Physician 1) 15 min. 8. Materials provided: Description of the homework assignments and a tracking sheet; physical exercise guide (part 1).
**Session 2:**
**Objectives:** To review the homework assignments and provide guidance when necessary; to reinforce the mental analgesia technique; to recognize how chronic pain affects participants’ lives; to understand the grieving process; to learn techniques for forgiving others and for self-acceptance; to identify low self-esteem and strengthen it; and to increase hope that pain can be controlled and well-being improved. **Content, techniques, facilitator, duration, and materials:** 1. Sharing the results of homework assignments. (Physician 1) 20 min. 2. Activity: Repetition of the mental analgesia technique. (T: meditation) (Physician 1) 10 min. 3. Presentation: Pain and its impact on the patient’s life: “You are not your pain.” (T: TCC, TAC, PRT) (Psychologist) 60 min. 4. Activity: Limiting labels. (T: role play) (Physician 1 and 5 patients) 15 min. 5. Presentation: Self-esteem. What is it? Activities: Reading “When I Truly Loved Myself”; self-esteem mirror affirmation (tools to increase self-confidence). (T: TAE) (Physician 1) 15 min. 6. Activity: Self-healing meditation. (T: meditation) (Physician 1) 10 min. 7. Activity: Forgiveness and self-forgiveness. Examples from experience. (T: Ho’oponopono, TAC) (Physician 1) 10 min. 8. Presentation: Physical exercise as a tool to reduce pain. Activity: Performance of physical exercises. (T: EHS, EP) (Physiotherapist) 35 min. 9. Talk: Examples of individuals with personal improvement experiences (a real patient from a previous workshop shares their experience). (T: MC) (patient contributor) 20 min. 10. Activity: Motivation for change. Life purpose. (Video of a person overcoming major limitations). (T: MC) (Physician 1) 5 min. 11. Homework assignments: The participants are taught two new tasks (Ho’oponopono technique and self-healing meditation) to practice during the week and are instructed to continue performing the Session 1 activities daily. (Physician 1) 10 min. 12. Materials provided: Description of the homework assignments; physical exercise guide (part 2).
**Session 3:**
**Objectives:** To review the homework assignments and provide guidance when necessary; to recognize how daily habits influence chronic pain; to identify habits that can improve pain control (nutrition, physical exercise, and sleep); to reinforce forgiveness of others and oneself; and to motivate behavioral change. **Content, techniques, facilitator, duration, and materials:** 1. Sharing the results of homework assignments. (Physician 1) 20 min. 2. Presentation: How personal conflicts influence pain. (T: ED, video) (Physician 1) 25 min. 3. Presentation: Promotion of healthy habits: General approach. Activity: Self-assessment of habits. (T: EHS, video, MC) (Physician 1) 20 min. 4. Presentation: Healthy eating. (Nutritionist and Nurse) (T: EHS, MC) 40 min. 5. Presentation: Sleep quality. (Nurse) (T: EHS) 30 min. 6. Activity: Physical exercise through dancing. (T: EP) (Physician 1) 10 min. 7. Activity: Metta meditation (focused on forgiveness). (T: meditation) (Physician 1) 10 min 8. Activity: Connecting with one’s inner self. (T: Reading “Who You Are”, song “I Will Wake You Up”) (Physician 1) 15 min. 9. Activity: Motivation for change, “Awakening the Senses.” (T: song, mindfulness, MC) (Physician 1) 10 min. 10. Talk: Experience shared by a patient from a previous workshop. (T: MC) (patient contributor) 20 min. 11. Homework assignments: The participants are given new tasks to complete during the week: improve one or two habits; identify what they say to themselves when they make a mistake and change their internal dialogue; and practice Metta meditation. They are instructed to continue performing the Session 1 activities daily. (Physician 1) 10 min. 12. Materials provided: Description of the homework assignments; and information on nutrition and sleep quality.
**Session 4:**
**Objectives:** To review the homework assignments and provide guidance when necessary; to understand the need to participate actively in improving one’s own health; to learn how to prepare for a medical consultation; to reinforce tools and advice that reduce pain and improve health; to address questions about the disease and/or treatment; to learn how to use visualization to achieve goals and improve the current situation; and to motivate change. **Content, techniques, facilitator, duration, and materials:** 1. Sharing the results of homework assignments. (Physician 1) 20 min. 2. Presentation: Active participation in my own recovery. How to cope with illness. (T: ED, MC) (Physician 3) 90 min. 3. Presentation: Creative visualization. (T: MC) (Physician 1) 15 min. 4. Activity: Physical exercise through dancing. (T: EP) (Physician 1) 15 min. 5. Activity: How to improve self-esteem: Video “You Are Not Good Enough,” about well-known individuals who persisted in pursuing their goals despite initial failures and ultimately achieved them. (T: MC, video) (Physician 1) 5 min. 6. Activity: Brief verbal survey to assess patients’ current status (whether they are controlling their pain and their current mood). (Physician 1) 10 min. 7. Talk: Experience shared by a patient from a previous workshop. (T: MC) (patient contributor) 20 min. 8. Activity: Self-esteem meditation. (T: meditation) (Physician 1) 10 min. 9. Activity: Self-connection through music: “The Eternal Sun.” (T: mindfulness, MC) (Physician 1) 10 min. 10. Homework assignments: The participants are taught a new task for the week, “Creative visualization,” and are instructed to continue performing the Session 1 activities daily. (Physician 1) 15 min. 11. Materials provided: Description of the homework assignments.
**Session 5:**
**Objectives:** To review the homework assignments and provide guidance when necessary; to review the tools, advice, and therapies presented and practiced during the workshop; to address questions about the tools; to collect the tracking sheets completed at home; to evaluate outcomes using scales and questionnaires through the app; and to thank patients for their participation and work. **Content, techniques, facilitator, duration, and materials:** 1. Sharing the results of homework assignments. (Physician 1) 15 min. 2. Presentation and discussion: Review of the tools explained during the workshop. Group sharing. Question-and-answer session. (Physician 1) 60 min. 3. Collection of the tracking sheets completed by patients during the workshop and provision of the recommendations guide. (Physician 1) 15 min. 4. Talk: Experience shared by a patient from a previous workshop. (patient contributor) 20 min. 5. Activity: Workshop evaluation and completion of assessment scales. (patients, with guidance from facilitators) 40 min. 6. Activity: Physical exercise through dancing. (T: EP) (Physiotherapist) 20 min. 7. Talk: Information about the upcoming follow-up dates. (Physician 1) 10 min. 8. Activities: Thanking patients for their trust in the workshop and encouraging them to continue applying and further developing the tools to control pain and cope better with illness; final closing and farewell. (Physician 1) 30 min. 9. Materials provided: Guide containing the tools presented during the workshop and additional advice; information on chronic pain patient associations located in the hospital’s catchment area.

T: Technique used; ED: pain education; TCC: cognitive behavioral therapy; TAC: Acceptance and Commitment Therapy; PRT: Pain Reprocessing Therapy; TAE: exercises to identify and improve low self-esteem; Ho’oponopono: technique for working on forgiveness; MC: motivation for change; EHS: healthy lifestyle education; EP: physical exercise. Note: PowerPoint presentations containing the content of the talks are available. Each facilitator developed their own presentations, which will remain unchanged throughout this study to ensure intervention fidelity. In some sessions, printed supporting materials are also provided to patients, as indicated in the table at the end of each session.

## Appendix B

### The Topic Guide for the In-Depth Interview

Before participating in the workshop:

- Description of the pain and how it affected the daily life of the participant.
- Description of how the participant carried out daily activities, including any activities they had stopped doing.
- Perceived self-esteem and self-care before the workshop.
- Relationships with others, including family, friends, work colleagues or people from other settings.

During participation in the workshop:

- Experiences and feelings elicited during the workshop.
- Relationships within the group.
- Difficulties in following the techniques during the workshop and in putting them into practice at home.
- Best and worst aspects of the workshop.

After participating in the workshop:

- Whether participants’ expectations were met.
- Difficulties encountered in applying the techniques in daily life.
- Which techniques or tools learned during the workshop have been most useful to you in managing your pain, and why?
- Which techniques or tools learned during the workshop have been most useful to you in improving your well-being and quality of life, and why?
- Were there any techniques that you found less useful?
- Perceived impact of what was learned on pain, analgesic use, self-esteem, self-care and daily life.
- Whether the workshop helped participants resume any activities they had stopped doing or had not been able to do for a long time. Turning point.
- Description of the participant’s future after the workshop, including perceived changes in their life.
